# Laser‐Induced Nanodroplet Injection and Reconfigurable Double Emulsions with Designed Inner Structures

**DOI:** 10.1002/advs.201900785

**Published:** 2019-07-03

**Authors:** Jin‐Kun Guo, Seung‐Ho Hong, Hyun‐Jin Yoon, Greta Babakhanova, Oleg D. Lavrentovich, Jang‐Kun Song

**Affiliations:** ^1^ Department of Electrical and Computer Engineering Sungkyunkwan University Suwon 16419 Republic of Korea; ^2^ Merck Performance Materials Ltd. Pyeongtaek 17956 Republic of Korea; ^3^ Advanced Materials and Liquid Crystal Institute Kent State University Kent OH 44242 USA; ^4^ Department of Physics Kent State University Kent OH 44242 USA

**Keywords:** double emulsions, laser injecting, liquid crystal droplets, marangoni effect, whispering gallery mode

## Abstract

Microfabrication of complex double emulsion droplets with controlled substructures, which resemble biological cells, is an important but a highly challenging subject. Here, a new approach is proposed based on laser‐induced injection of water nanodroplets into a liquid crystal (LC) drop. In contrast to the conventional top‐down microfluidic fabrication, this method employs a series of bottom‐up strategies such as nanodroplet injection, spontaneous and assisted coalescence, elastically driven actuation, and self‐assembly. Each step is controlled precisely by adjusting the laser beam, interfacial tension, and its gradients, surface anchoring, and elasticity of the LC. Whispering gallery mode illumination is used to monitor the injection of droplets. A broad spectrum of double emulsions with a predesigned hierarchical architecture is fabricated and reconfigured by temperature, laser‐induced coalescence, and injection. The proposed bottom‐up method to produce customized microemulsions that are responsive to environmental cues can be used in the development of drug delivery systems, biosensors, and functional soft matter microstructures.

Micro‐ and nanofabrication of solid materials achieved a great success in semiconductor industry by incorporating complex electronic functions into microscale devices. A similar strategy can in principle be applied to soft matter,^1^, ^2^, ^3^, ^4^ including liquid crystals (LCs)^5^, ^6^ to fabricate multilevel hierarchical structures with broad applicability in drug delivery,^7^ food processing,^8^ cosmetics,^9^ and microchemistry.^10^ However, microfabrication in liquid and soft systems is severely constrained by their peculiar properties such as fluidity, zero or weak resistance to shear, surface tension, and diffusion.^1^, ^11^ These factors significantly limit the spectrum of microfluidic designs, so that only relatively simple structures could be produced, such as spheres of one fluid embedded in a sphere of another fluid. Recently, Solodkov et al.^5^ demonstrated that double emulsions with multiple water droplets in an LC drop can be produced by combining syringe pumps and controlled shaking. These emulsions adopt an equilibrium shape controlled by the elasticity of the LC. Meanwhile, living cells comprised of various soft matter elements show sophisticated out‐of‐equilibrium architectures, complexity, adaptability, and responsiveness that exceed by far the man‐made microfluidic crafts. While the artificial microfluidics employs mostly top‐down techniques such as coaxial microcapillary injection,^12^, ^13^ nature designs soft architectures by bottom‐up approaches, with intertwined crowding effects, phase separation, partial orientational and positional order, selective membrane permeability, diffusion, and self‐assembly. This difference suggests that integration of the bottom‐up approaches into microfluidics might open the door for qualitatively new strategies in the microscale design of hierarchical soft matter.

Here, we present a bottom‐up strategy of microfabrication of double emulsions representing hierarchical dispersion of microdroplets of water in a thermotropic oil‐like nematic LC. The LC volume itself is a spherical drop placed in an aqueous solution of a surfactant that sets perpendicular orientation of the LC molecules at the interface. The microfabrication is initiated by laser‐induced injection of nanoscale water droplets at a preselected site of the LC–water interface; no syringe is required. The injected droplets grow, move, and interact with the background orientational field of the LC to form complex structures supported by the elasticity of the LC host. By adjusting the laser beam and irradiation site, temperature, and concentration of ingredients, we fabricate three‐dimensional (3D) double emulsion architectures and demonstrate how to reconfigure them. Among these structures are chiral tetrahedral assemblies and structures that release the water droplets from the LC bulk back into the environment. The physical effects involved sequentially and collaboratively in this bottom‐up fabrication are multifaceted, ranging from laser‐triggered modification of LC and LC–water interface,^14^, ^15^ intrinsic elasticity, and anisotropic interfacial energy of the LC,^16^, ^17^ to formation of topological defects mediating long‐range colloidal interactions in LCs.^18^, ^19^, ^20^

The host nematic drops of a radius *R* ≈ 50–150 µm are created by dispersing the LC (E7 + 0.3 wt% coumarin‐6, see the Experimental Section) in water. The dispersion is stabilized by a surfactant sodium dodecyl sulfate (SDS), with concentration in the range of 10 × 10^−3^ to 320 × 10^−3^ m, which is higher than the critical micellization concentration 8.9 × 10^−3^ m, to assure their presence at the LC–water interfaces. SDS sets perpendicular anchoring of the LC molecules at the surface of the LC drops, so that the director field (black dash line) in the bulk is radial, with the point defect (red dot), the so‐called radial hedgehog, in the center of the drop (**Figure** **1**a).^21^

**Figure 1:**
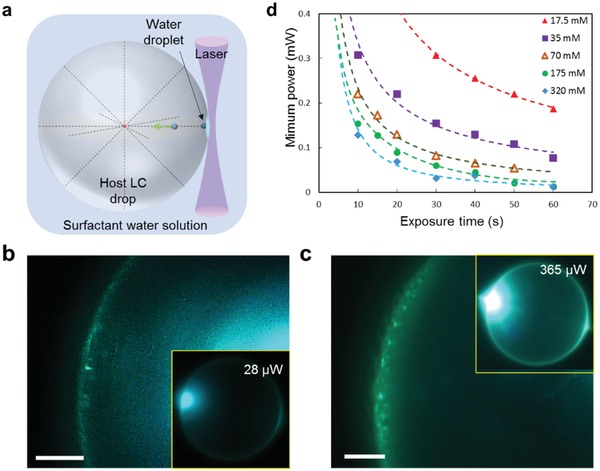
Laser‐induced injection of water nanodroplets into an LC drop with radial director. a) Scheme. b,c) Exposure to 28 µW (b) and 365 µW (c) beam inject droplets visualized by WGM illumination. d) Minimum beam power and exposure time needed for droplet injection at various SDS concentrations. Scale bars: 20 µm.

The water nanodroplets are injected into the host LC drop by irradiation of a laser beam of a small diameter (10–60) µm focused at the LC–water interface (Figure 1a and the Experimental Section). The injection is observed at an extraordinary weak laser powers, starting with 28 µW, for illumination duration of 50 s. Since the injected droplets are smaller than the resolution limit of optical microscopy, we use a whispering gallery mode (WGM) illumination of the LC drop with an added fluorescent dye.^22^ The laser beam of power 28 µW was first used to inject the guest droplets, and then the beam was weakened to 10 µW and repositioned to the opposite side of the LC drop to trigger WGM and visualize the droplets, Figure 1b,c.

A higher laser power produces a higher number of guest droplets, as illustrated in Figure 1c for a 365 µW laser beam with 20 µm diameter focused for 20 s. The WGM observations suggest a rough estimate 2*a* ≈ (0.4 − 1.2) µm for the diameter of the droplets (Figure S1, Supporting Information); apparently, smaller droplets are also present but hard to detect. Note here that surfactant‐mediated instabilities with detachment of nanodroplets as small as 200 nm in diameter are known as “tip streaming,“caused by strong flows at the interface between two immiscible isotropic fluids.^23^

Figure 1d illustrates what minimum power and exposure time is needed for droplet injection at various surfactant concentrations. For 320 × 10^−3^ m SDS solution, 15 s exposure of 100 µW laser beam is enough to inject a water droplet, but for 17.5 × 10^−3^ m SDS solution, six times more energy (that is, 30 s exposure of 300 µW) is required. This result agrees well with the temperature dependence of the interfacial tension σ(*T*) in **Figure** **2**a, which shows an increasing slope at higher SDS concentration, and suggests that the interfacial tension gradients induced by laser heating can be responsible for the injection.

**Figure 2:**
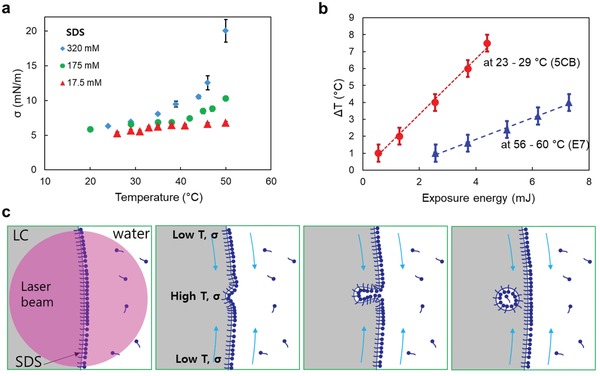
LC–water interface. a) Interfacial tension of the nematic E7 increases with the temperature for various SDS concentrations. b) Laser‐induced temperature increase in 5CB and E7 drops (see the Experimental Section). The slopes of the fitted lines are 1.68 and 0.63 °C mJ^−1^, respectively. c) Plausible injection mechanism.

To determine the local temperature *T* increase, we monitored the nematic‐to‐isotropic phase transition triggered by the laser beam (see the Experimental Section) and found that a 20 s exposure of a 100 µW beam increases *T* by 3 and 1 °C, when the ambient temperature is 23 and 60 °C, respectively (Figure 2b). Heating causes an increase of the interfacial tension σ on the order of 10^−4^ to 10^−3^ J m^−2^, depending on the concentration of SDS, Figure 2a,^24^ similarly to the data obtained for other materials.^25^ One can thus estimate the Thomson–Marangoni tangential stress ∇*_||_σ* ≈ Δσ/L, where *L* is the length scale over which *T* varies. Even if one overestimate as being larger than the beam cross‐section, *L* ≈ 100 µm, the resulting ∇*_||_σ* is still very large, (1–10) N m^−2^, at least an order of magnitude higher than the stress reported in ref.^26^ to cause splitting of water droplets. Hence, the laser‐induced local heating can cause instabilities of the interface that result in guest droplet injection. Although the concrete mechanism needs further studies, a plausible scenario is illustrated in Figure 2c. The laser‐illuminated site heats up, causing a higher σ. Material flows from colder regions toward the illuminated spot produce a bulge that transforms into a small injected water droplet. This scenario is consistent with the model of interfacial instability,^27^ according to which detachment of small droplets from an interface of two immiscible fluids occurs at a local convergence of streamlines near the zero‐vorticity point; in our case, this zero‐vorticity point is produced by the focused laser beam. The droplets contain a substantial amount of SDS, since they usually exhibit perpendicular surface alignment of the director around them, as discussed below.

The injected nanodroplets merge into larger ones until reaching a certain size, **Figure** **3**a. Droplets of isotropic fluids in an immiscible isotropic medium coalesce to reduce the interfacial area. In an LC, however, the coalescence of isotropic droplets can be arrested, as demonstrated by Loudet et al.^28^ Since the elastic energy of an LC around a spherical inclusion of a radius *a* scales as ≈*Ka*, where *K* is the Frank elastic constant, and the surface anchoring energy scales as *Wa* ^2^, where *W* is the anchoring coefficient, the behavior of the droplets depend on whether *a* is smaller or larger than the anchoring extrapolation length ξ = *K*/*W*, typically on the order of a micrometer. When *a* << ξ, the water droplet does not distort the LC, as the elastic energy would be higher than the surface anchoring energy. When *a* > ξ, however, the director starts to deform around the droplet in order to satisfy the perpendicular anchoring conditions. The radial director field in the immediate vicinity of the droplet is supplemented a point‐defect, the so‐called hyperbolic hedgehog that connects the director fields near the water droplet and away from it (Figure 3b). We observe these point defects when *a* ≥ 1 µm. Elastic repulsion between the droplets with hedgehogs prevents their coalescence and arrest their growth at *a* ≈ ξ, Figure 3c.^28^

**Figure 3:**
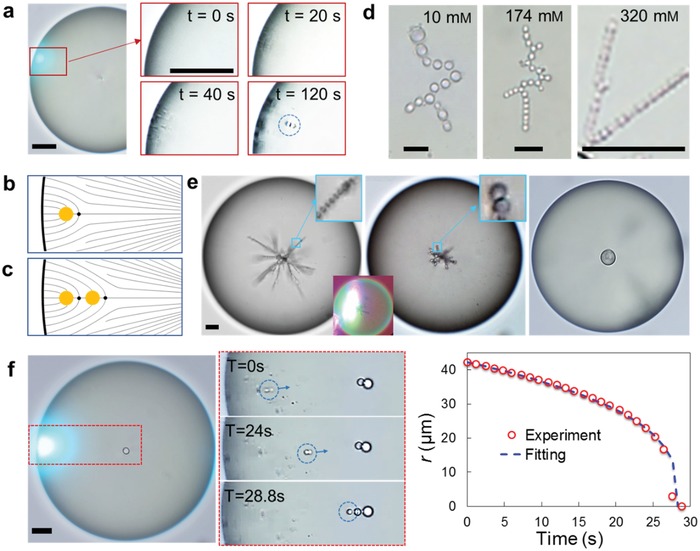
Injection and dynamics of water droplets. a) Coalescence of injected nanodroplets. b) The director field in the host LC drop with a large *a* ≈ ξ water droplet and accompanied hedgehog. c) Coalescence of droplets is prevented by the hedgehog between them. d) The injected water droplets at varying SDS concentrations (10 × 10^−3^, 174 × 10^−3^, and 320 × 10^−3^ m) of diameter 2*a* = 3.4, 1.8, and 0.9 µm, respectively. e) Coalescence of water droplets triggered by 1 mW laser irradiation. f) Elastically mediated acceleration of a water droplet from the periphery to the center of the host LC drop. Scale bars: 10 µm.

As follows from the discussion above, coalescence can be controlled by modifying *K* and *W*. The surfactant concentration directly influences *W*,^25^ and thus the size of guest droplets. For SDS concentrations of 10 × 10^−3^, 174 × 10^−3^, and 320 × 10^−3^ m, the droplets grow to 2*a* = 3.4, 1.8, and 0.9 µm, respectively, Figure 3d. Interestingly, droplets in a system with a higher SDS concentration assembly into linear chains, while the droplets at low concentrations of SDS also show slanted assembly observed for tangentially anchored droplets,^29^ Figure 3d. The results suggest that *W* increases with SDS concentration.^30^ Ambient temperature can also be used as a control parameter, since the diameter 2*a* decreases with the increasing temperature, relatively slow in the range from 18 to 35 °C, and then rapidly in the range from 35 to 60 °C (Figure S3, Supporting Information); this effect is mediated by temperature dependencies of the LC properties. Enlargement of injected droplets can also be achieved by heating with a strongly focused laser beam that melts the LC locally and thus triggers droplets coalescence, Figure 3e. This technique allows us to produce a single large “core“water droplet in the center of the LC drop that satisfies the perpendicular anchoring condition and does not need a hyperbolic hedgehog, Figure 3e.

When guest droplets near the LC–water interface grow sufficiently large, *a* ≈ ξ, they become mobile and move with acceleration toward the center of the host, Figure 3f. We describe these dynamics as follows. According to the theory of Pergamenshchik,^31^, ^32^ the elastic interaction potential of a guest microsphere within a background deformed director **n̂(r)**, which represents a pure splay in our case, writes

(1) $$U_{d}=-8\pi \beta Ka^{2}(\hat{p}\cdot \hat{n})\;\nabla \cdot \hat{n}$$

where β is the numerical coefficient that depends on *W*, $\hat{p}$is the unit vector directed from the hyperbolic hedgehog toward the microsphere. The hedgehog is always located on the side of the microsphere that is closer to the center of the nematic drop to fit the underlying splay.^31^ For splay, $(\hat{p}\cdot \hat{n})\;=\;1$, and Equation (1) reduces to *U*_d_ = −16*πβa* ^2^/*r*, where *r* is the radial coordinate of the droplet. The corresponding driving force *F*_r_ = −∂*U*_d_/∂*r* = 16*πβKa* ^2^/*r* ^2^ is balanced by the viscous drag, *F*_drag_ = −*6πaη_ǁ_v*, where *v* = d*r/*d*t* is the velocity of the droplet, and *η_ǁ_* is the viscosity of LC. This balance yields the radial position of the water droplet as a function of time

(2) $$r={\left(R^{3}-8\beta Kat/\eta_{\left|\right|}\right)}^{1/3}$$

This theoretical prediction fits the experiment in Figure 3f well for *R* = 42 µm, *a* = 1.6 µm, and the typical *η_ǁ_ =* 10 mPa s, and *K* = 10 pN.^31^ The fitting yield β ≈ 0.21, close to the value 0.20 found for colloids driven by patterned director.^31^

Freshly injected nanodroplets, *a* ≤ ξ, move toward the LC host center very slowly or do not move at all, which can be explained following Voloschenko et al.^33^ A small particle at the periphery of an LC drop, *a* << *R*, eliminates splay distortions in a volume ≈*a* ^3^ and reduces the elastic energy by ≈*Ka* ^3^/*R* ^2^, which for *K* ≈ 10 pN, *a* ≈ 1 µm, and *R* ≈ 50 µm is very small, ≈4 × 10^−21^ J, on the order of the thermal energy *k*_B_ *T*. The corresponding driving force is too weak to transport small droplets toward the center of the LC drop. If Brownian diffusion brings the small droplet closer to the center, *r* << *R*, then the elastic gain ≈*Ka* ^3^/*r* ^2^ becomes larger and this droplet can accelerate toward the center, as observed experimentally.

The proposed method of double emulsion formation offers a unique possibility for a controlled assembly. All water droplets of a radius *a* ≈ ξ move toward the center of the host LC drop because of the elastic attraction described above, Figure 3f. The structural dipoles **p** of these droplets are attracted head‐to‐tail,^16^ remaining everywhere parallel to the radius‐vector of the LC drop, so that the droplets form polar linear chains (Figures 3c and 4a). By choosing the location of droplets injection, one can construct an extraordinarily rich variety of complex structures, **Figures** **4** and **5**, as described below.

**Figure 4:**
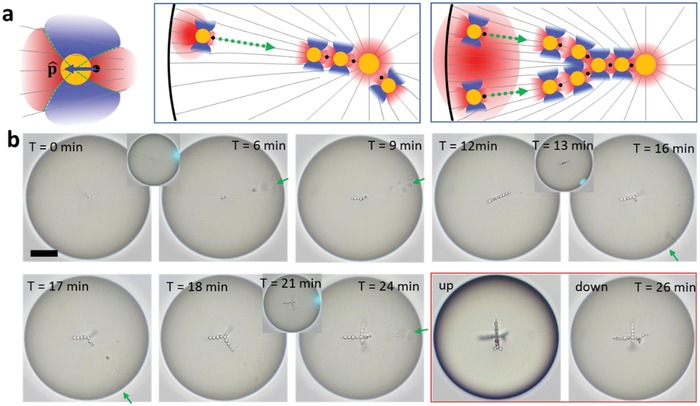
Design of 3D architectures. a) Sectors of attractive (red) and repulsive (blue) elastic interaction of droplets with *a* ≈ ξ within a radial director field (left). The central core water droplet attracts radially growing chains (middle). At large distances from the core, radial chains can split (right). b) Tailored injection and guided assembly of water droplet in a host LC drop in 70 × 10^−3^ m SDS solution. A 10 µm diameter 60 µW beam irradiates the interface for 20 s (inset at *T* = 0 min). The injected nanodroplets coalesce into larger one, and form a chain of nine droplets extended toward the exposure spot (green arrow). Then the laser‐injection site is moved to different locations (green arrows at *T* = 13 min and 21 min) to grow new branches and form triangular and tetrahedral structures. Two images at *T* = 26 min were taken at different focal planes of the microscope. Scale bars: 20 µm.

**Figure 5:**
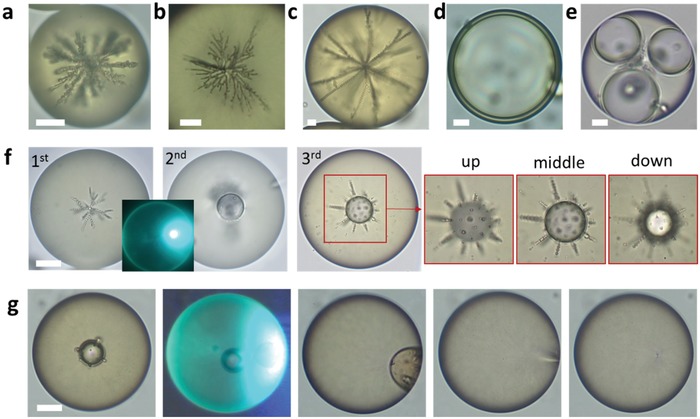
Reconfiguration of 3D architectures. a,b) Self‐assembled high‐order branched assemblies. c) The branches reach the surface of the host LC drop. d,e) LC shell with a central water core produced by laser‐trigged coalescence of guest droplets. f) Assembly architecture is reconfigurable by laser‐induced coalescence and self‐assembly of small guest droplets. The three expanded images correspond to different focal planes of the microscope. g) Release of droplets through a high‐power laser exposure (60 mW, 20 s) that melts the LC. Scale bars: 10 µm.

Figure 4b illustrates a sequential build‐up of a double emulsion in an LC host drop by selecting the locations and number of laser‐injected water nanodroplets. The laser beam is focused for 20 s and injects nanodroplets that coalesce into nine microdroplets that form a linear chain. Then the injection point and the duration of irradiation are changed (insets at *T* = 13 min and 21 min) to introduce new radial branches. This way, one can build linear, triangular, and tetragonal structures, Figure 4. The linear chains that pass through the center of the drop are straight, since the two radial subsections repel each other elastically.^5^ For the same reason, the triangular structure is practically flat, with the angle between the chains being 120° ± 10° (Figure 4b at *T* = 18 min). The branches in the tetrahedral structure in Figure 4b (*T* = 26 min) are designed with a different number of droplets, thus the angle between them deviates from its “symmetric“tetrahedral value 109.5°. Moreover, since four chains emanating from the central core are different from each other, this structure is chiral, i.e., nonsuperposable on its mirror image, reminding chiral organic molecules with asymmetric carbon center. As the branches extend, they can split (see Figures 4a,b and 5a–c). The reason is that as *r* increases, the available area 4*πr* ^2^ becomes large and there is space to place more mutually repelling water droplets with sufficient separation from each other. The splitting is especially clear in structures with a large number of droplets, Figure 5a–c.

Solodkov et al. demonstrated that water–LC emulsions created by microfluidics and shaking are “self‐assembled into tetrahedral configurations almost exclusively and lower order configurations are possible but rarely observed.”^5^ Apparently, the exclusively stable status of the tetrahedral states is helped by shaking that eliminates metastable states. Our bottom‐up approach with the controlled injection sites and size of the injected droplets allows us to create a much wider spectrum of architectures, including those of low order in Figure 4b and high order in Figure 5a–c,f. These high‐order structures are produced by using a wide beam spot that generates multiple guest droplets simultaneously and in close proximity of each other, as illustrated in Figure 4a. All the assemblies in Figures 4b and 5a–c,f remain stable for at least one week of observation.

Using the laser beam to force coalescence, we can control the ratio *a*_c_/*a* of the radius *a*_c_ of the central drop and the radius of the droplets in the attached branches. According to the theory,^5^ the ratio *a*_c_/*a* is the main factor defining the number of branches emanating from the central core: since the chains of droplets repel each other and attract to the central core, a larger central core allows more chains to be attached. The structure in Figure 5c with *a*_c_ ≈ 6.3 µm, 2*a* ≈ 3.5 µm, and *a*_c_/*a* ≈ 3.6 shows 8  ±  2 branches while Figure 5b with *a*_c_ ≈ 3.5 µm, but much smaller satellite droplets, 2*a* ≈ 0.9 µm and *a*_c_/*a* ≈ 7.7, shows a much higher number of branches, 25 ± 5; uncertainties are associated with the fact that some chains emanating from the core droplet start to branch very early. Repulsion forces between chains become weaker at smaller *a*,^34^ and it makes it possible to form more chains, compare Figure 5b to Figure 5c. The number of brushes in these figures correlates with the theoretical expectations,^5^ of about 12 branches for *a*_c_/*a* ≈ 3.6 and 33 branches for *a*_c_/*a* ≈ 7.7.

Laser‐assisted coalescence can be used to create an LC shell with a single large water droplet at the core (Figure 5d) or a few large droplets (Figure 5e) near the center. It can also reconfigure the branched structures, Figure 5f. The branched cluster at step 1 was fused into a large drop in step 2, by exposure to a 1 mW laser beam for 5 min. In step 3, by injecting additional small droplets of diameter 2*a* ≈ 1 µm, we fabricated a complicated structure with 32 chains, attached to a very large central core with *a*_c_ ≈ 11 µm and *a*_c_/*a* ≈ 21, Figure 5f.

In applications such as drug delivery, the release of guest droplets is highly important. In our approach, such a release can be achieved simply by exposing the LC drop to a high‐power laser beam (60 mW, 20 s) that melts the LC into the phase, Figure 5g. In the isotropic phase, the LC elasticity is absent, thus the host drop loses its trapping ability.^35^ As a result, the guest droplets escape from the host (Figure S4, Supporting Information). The direction of droplet release can be selected by exposing a focused laser at the target position so as to induce the nematic to isotropic transition only locally, as in Figure 5g.

In conclusion, we demonstrate a facile and precise method to inject submicrometer tiny water droplets into a host LC drop using a weak laser beam. These tiny water droplets spontaneously coalesce and grow, until they reach the size comparable to the anchoring extrapolation length. Once of this size, the droplets distort the director field around them and show elastically mediated long‐range interactions of attractive and repulsive nature with other droplets and with the background splay deformations of the director in the host LC structure. Anisotropic interactions of the water droplets produce double emulsion clusters with unprecedented complexity and flexibility. By choosing the location and other parameters of the droplets laser injection, one can construct various hierarchical architectures that remain stable or metastable thanks to the elastic interactions mediated by the LC medium. In particular, we demonstrated tetragonal structures of chiral symmetry. Morphology of the structures can also be controlled by adjusting the laser parameters, such as power and beam diameter. Potentially, it can also be controlled by the wavelength of laser irradiation, through the adjustment of spectral sensitivity of the dye used to enhance light absorption. Surfactant chemistry and concentration can also be used as a control parameter. The proposed method of the bottom‐up design of complex architectures is enabled by a rich spectrum of intertwined phenomena including bulk elasticity of LC, anisotropic interfacial energy, and laser‐induced surface gradients.

The proposed approach can be further advanced for various applications such as drug delivery systems,^36^ biosensors,^37^ and other functional soft matter microstructures.^5^ Precise loading and encapsulating biomedical materials with controlled volume fraction in carrier drops with biocompatibility, and triggered releasing of chemicals are highly demanded technologies in drug delivery.^4^, ^36^, ^38^ In addition, one can inject guest droplets with different chemicals sequentially by replacing the surrounding solution, and fabricate stable double emulsions with multicomponent guest droplets in a single host drop.^39^ The multicomponent guest dropelts can be merged by external stimulti such as laser exposure, thermal energy, and pressure, and the mergence may result in chemical reaction or change in color. This can be a promising route for biosensor, environmental sensor, and drug delivery systems.

## Experimental Section

*Materials*: Anionic surfactant SDS and fluorescent dye coumarin‐6 were purchased from Sigma‐Aldrich (Korea). Liquid crystals MLC‐7026‐000, E7, and 5CB were obtained from Merck (Korea). Coumarin‐6 was used as a dopant (0.1–0.3 wt%) for enhancing the light absorption and fluorescent measurements. In most experiments, a well‐known LC mixture, E7, was used. E7 doped with 0.3 wt% coumarin‐6 has nematic to isotropic phase transition temperature (*T*_NI_) of about 60.5 °C and the average elastic constant 13 pN. For droplet injection in Figures 4 and 5a–f, MLC‐7026‐000 with 0.1 wt% coumarin‐6, which has *T*_NI_ of about 80 °C and the average elastic constant of 15 pN, was used. MLC‐7026‐000 shows a slower injection speed, and makes it easier to inject droplets one by one. 5CB was used to detect the laser‐induced temperature increase at room temperature, since it has relatively low *T*_NI_ (≈30 °C).

*LC Drops*: To impose perpendicular surface anchoring, solutions of SDS was used in deionized water. The mixture of the LC and coumarin‐6 (0.1 or 0.3 wt%) was sonicated for 0.5 h and stirred for 10 h to make homogeneous mixtures. Then, the LC was emulsified in water by simply adding the LC mixture in water and shaking the bottle for 1 min. The resulting emulsion represented LC drops dispersed in water and stabilized by SDS. The emulsion was injected into a cell formed by two glass plates separated by 150 µm.

*Laser Beams*: A 405 nm diode laser (Dragon laser, China) with adjustable power in the range from 1.23 to 60.6 mW was used. By inserting different pinholes between the laser and the microscope with a ×40 objective lens, the spot diameter at the sample was selectively switched among 10, 20, 40, and 60 µm. The laser power was controlled from 10 to 128 µW for 10 µm spot size, from 82 to 365 µW for 20 µm spot size, from 0.36 to 1.6 mW for 40 µm spot size, and from 13 to 60 mW for 60 µm spot size. The power of laser beam was varied depending on the purpose of experiments; the power was 10 µW for WGM illumination, 28 to 365 µW for the injection of guest water droplets, 1 mW for the coalescence of guest water droplets, 60 mW for the triggered release of guest water droplets.

*Polarized Optical Microscopy (POM)*: The POM analysis of textures was performed using an optical microscope BX51 (Olympus, Japan). The pictures were captured and the droplet motion was tracked (25 frames per second) by a CCD camera (TrueChrome HD, Tucsen, China) mounted on the eyepiece of the microscope.

*Interfacial Tension Measurement*: The interfacial tension was measured using the home‐built pendant drop tensiometer with CMOS camera (Figure S2, Supporting Information).^24^ The pendant drops of LC (3.75 mL) were formed in a temperature controlled SDS water solution with different concentration using a 22 gauge needle.^40^ The captured data are analyzed using open source software.^41^

*Laser‐Induced Local Temperature Increase*: The experiment was performed by observing the laser‐induced phase transitions of dye‐added 5CB and E7 drops (0.3 wt% coumarin‐6). By finding the minimum temperature (*T*_MIN_) of the sample to have local isotropic phase after 20 s exposure of laser beams with varying power, the local temperature increase (Δ*T*) was calculated as Δ*T* = *T*_NI_–*T*_MIN_.

## Conflict of Interest

The authors declare no conflict of interest.

## Supporting information

Supplementary
