# Supplementary material for: Laser‐Induced Nanodroplet Injection and Reconfigurable Double Emulsions with Designed Inner Structures
Source: Adv Sci (Weinh). 2019 Jul 3;6(17):1900785. doi: 10.1002/advs.201900785 (PMC6724358; doi:10.1002/advs.201900785)
Supplement: Supplementary file 1 — Supplementary [file ADVS-6-1900785-s001.pdf]

Copyright WILEY-VCH Verlag GmbH & Co. KGaA, 69469 Weinheim, Germany, 2019.

ADVANCED SCIENCE
Open Access

Supporting Information

for *Adv. Sci.,* DOI: 10.1002/advs.201900785

Laser-Induced Nanodroplet Injection and Reconfigurable Double Emulsions with Designed Inner Structures

Jin-Kun Guo, Seung-Ho Hong, Hyun-Jin Yoon, Greta Babakhanova, Oleg D. Lavrentovich, and Jang-Kun Song**

Copyright WILEY-VCH Verlag GmbH & Co. KGaA, 69469 Weinheim, Germany, 2019.

Supporting Information

Laser-induced Nanodroplet Injection And Reconfigurable Double Emulsions With Designed Inner Structures

Jin-Kun Guo, Seung-Ho Hong, Hyun-Jin Yoon, Greta Babakhanova, Oleg D. Lavrentovich and Jang-Kun Song**

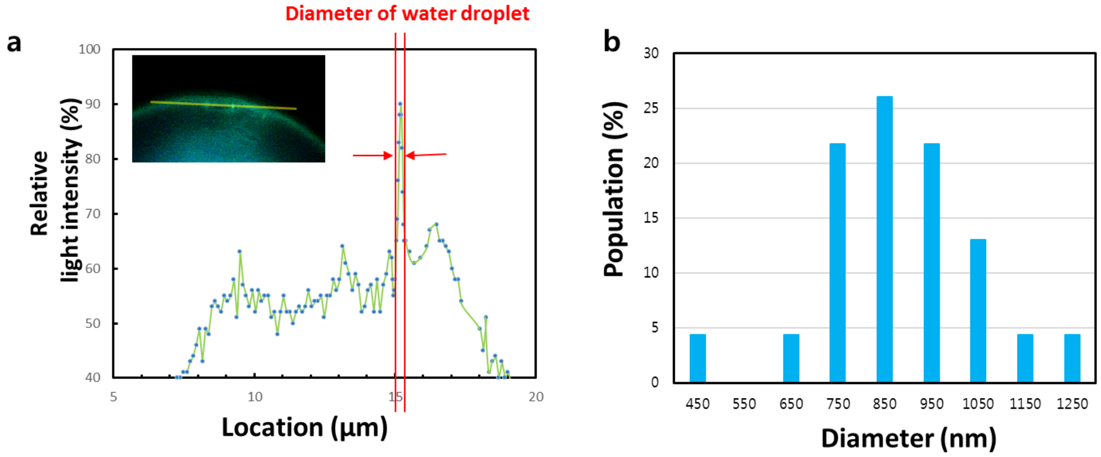

Figure S1. The diameter distribution of injected water droplets. a) By analyzing the WGM images, the diameter of droplets was estimated from the FWHM (full width at half maximum) of the intensity profile of spot highlighted by WGM fluorescent light. b) The diameter distribution of injected water droplets was measured after exposure to a laser beam with 365μW for 20s. In the inset in part (a), the auxiliary yellow line is drawn across the injected water droplet (bright spot) and the boundary of the host LC droplet in the WGM texture. By plotting the light intensity at each pixel along this line, normalized by the maximum intensity, one estimates the droplet diameter as the FWHM of the intensty peak.

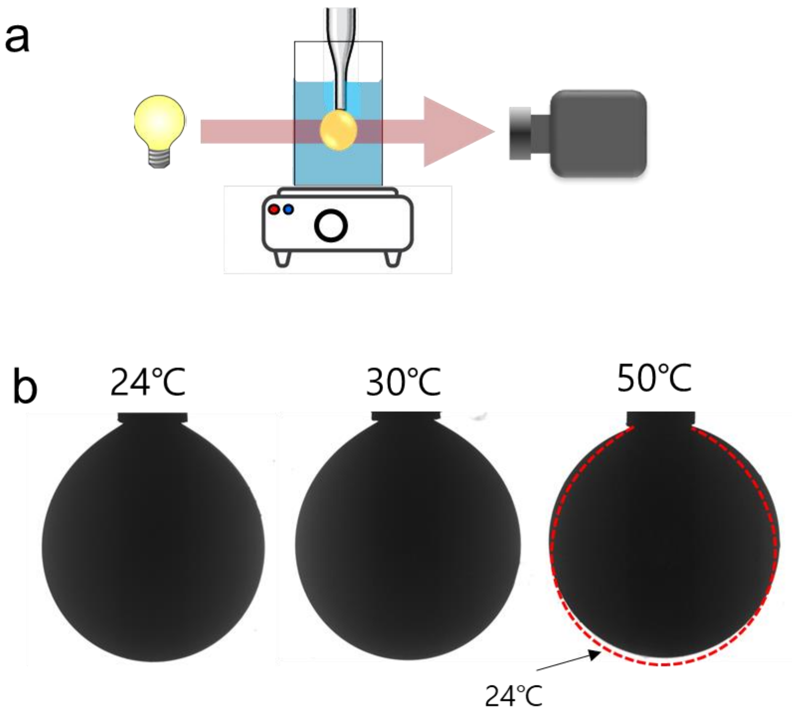

Figure S2. Interfacial tension measurement of LC droplet in surfactant-water solution. a) A simple pendant drop tensiometer was built by combining a complementary metal-oxide semiconductor camera and temperature controllable water bath. The volume of LC drop was 3.75 ml. b) The shape of E7 drop changes with temperature. The shape at low temperature is elongated vertically, and its shape becomes more spherical on increasing temperature, indicating the increasing surface tension. The captured images were analyzed using an open-source software provided by Daerr and Mogne,[1] to calculate the surface tension. The measured interfacial tension of LC (E7) drops is shown in Figure 2a.

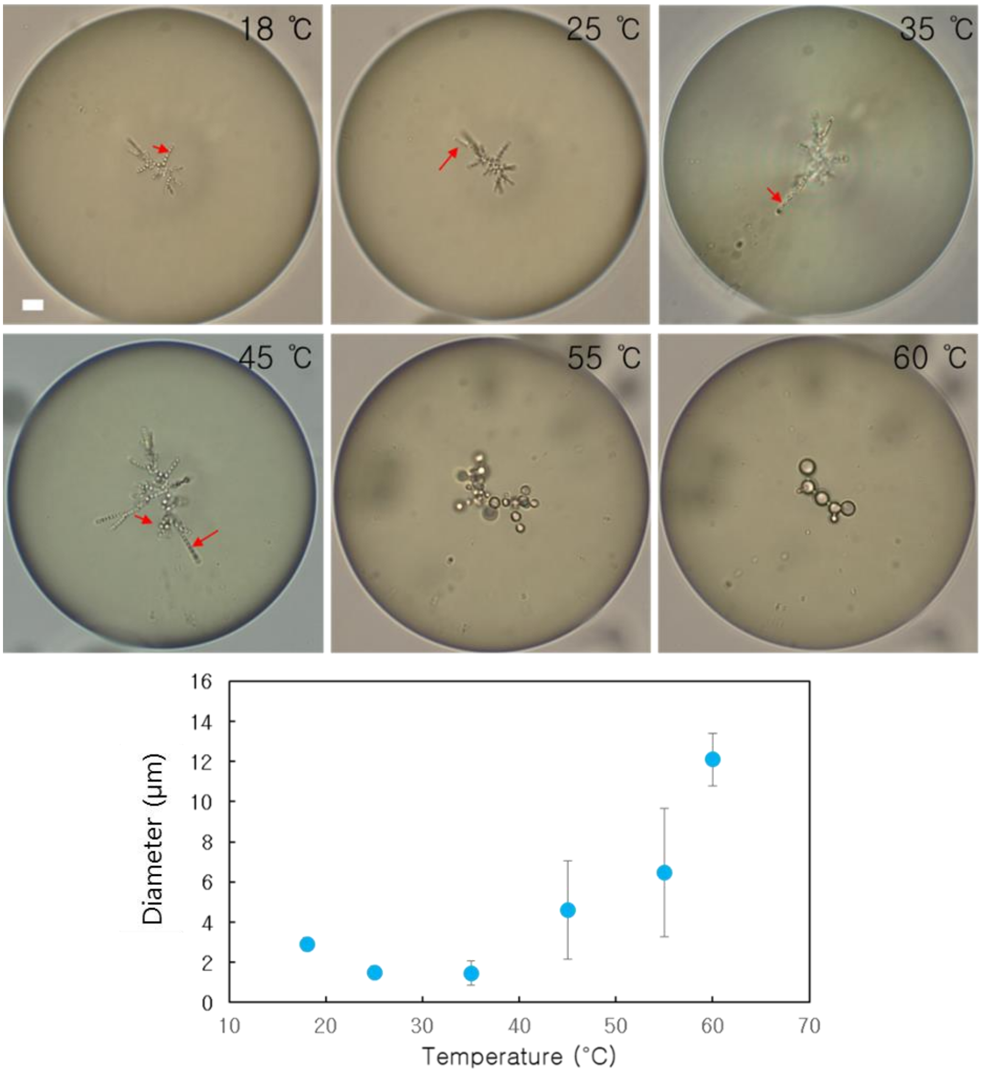

Figure S3. The diameter of guest droplets as a function of temperature, dispersed in a drop of the liquid crystal MLC-7026-000. At each temperature, additional droplets were injected and their diameter was measured after 10 min of stabilization. Up to 35 °C, the mean diameter of injected guest droplets slightly decreased with temperature, but above 35 °C, the guest droplets merge and grow. When the temperature increases further towards the clearing temperature, most of the droplets are merged. Scale bars, 20 μm.

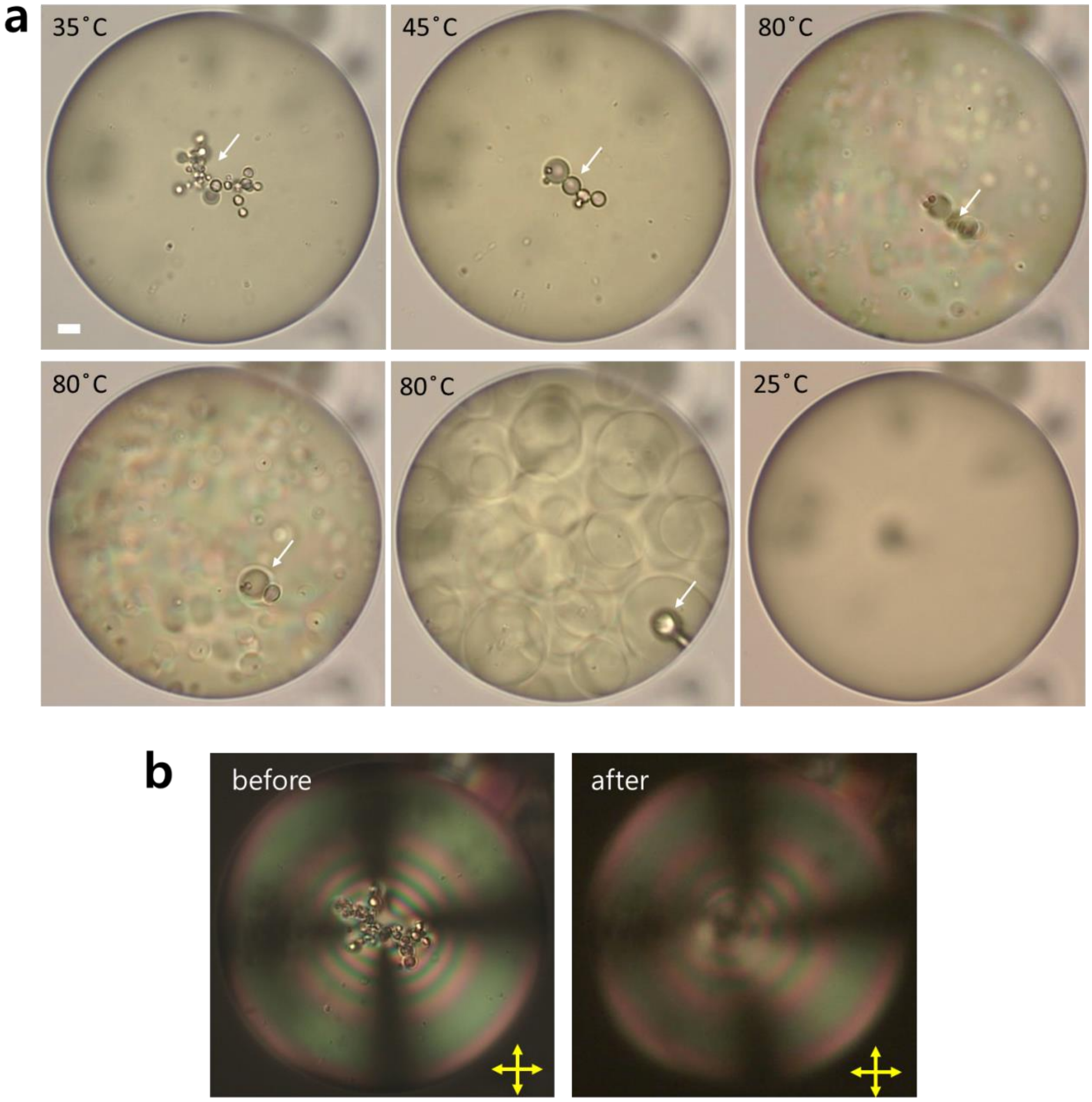

Figure S4. Thermal release of guest droplets from a host LC drop (MLC-7026-000). a) When the temperature reaches near the isotropic transition temperature, the hedgehog defect in the center is collapsed. As a result, the guest droplets are not trapped in the center, and drift randomly. When the guest droplets reach the boundary, they escape from the host drop (the last image). Arrows indicate the location of water droplet. b) POM images under crossed polarizers (yellow arrows), before and after the droplet release. Scale bars, 20 μm.

Reference

[S1] A. M. Daerr, A., J. Open Source Softw. **2016**, 4, e3.
